# Gancao (*Glycyrrhizae* Radix) provides the main contribution to Shaoyao-Gancao decoction on enhancements of CYP3A4 and MDR1 expression via pregnane X receptor pathway *in vitro*

**DOI:** 10.1186/s12906-018-2402-7

**Published:** 2018-12-29

**Authors:** Dandan Feng, Tao Tang, Rong Fan, Jiekun Luo, Hanjin Cui, Yang Wang, Pingping Gan

**Affiliations:** 1 Institute of Integrative Medicine, Xiangya Hospital, Central South University, Changsha, 410008 People’s Republic of China; 2 Department of Oncology, Xiangya Hospital, Central South University, Changsha, 410008 People’s Republic of China

**Keywords:** Shaoyao Gancao decoction, Pregnane X receptor, Cytochrome P450 3A4, Multidrug resistance protein 1, Traditional Chinese medicine

## Abstract

**Background:**

Chinese herbal formula Shaoyao Gancao decoction (SGD) is often used as an adjuvant with chemotherapeutic agents to treat cancer. Due to the herb-drug interactions, the alternations of drug metabolic enzyme and drug transporters induced by SGD deserve to be explored. We aimed to investigate the effect of SGD on the pregnane X receptor (PXR)-mediated transcriptional regulation of cytochrome P450 3A4 (CYP3A4) and drug transporter multidrug resistance protein 1 (MDR1) *in vitro*. Besides, we assessed the contribution of constituent herbs to SGD on the regulation of CYP3A4 and MDR1.

**Methods:**

The dual luciferase reporter gene system containing the hPXR expression plasmid and the reporter gene plasmid of CYP3A4 or MDR1 was co-transfected to HepG2 and Caco2 cells. Luciferase activities were determined using a Dual-luciferase reporter assay kit. The gene expression of CYP3A4 and MDR1 in the hPXR-transfected LS174T cells were assessed by real-time qPCR. Finally, the contribution of constituent herbs from SGD was evaluated.

**Results:**

SGD, Shaoyao and Gancao concentration-dependently increased promoter activities of CYP3A4 and MDR1 *in vitro*. Moreover, SGD, Shaoyao and Gancao up-regulated CYP3A4 and MDR1 mRNA in hPXR-transfected LS174T cells. As the herbal constituent of SGD, Gancao possesses significantly higher levels of metabolic enzyme and drug transporters compared with Shaoyao.

**Conclusion:**

SGD tends to enhance CYP3A4 and MDR1 expression via PXR pathway, especially Gancao provides the main contribution. This study highlights a potential *in vitro* mechanism for SGD on the regulation of drug metabolic enzymes and drug transporters.

## Background

Chinese herbal medicine becomes the main adjuvant combined with chemotherapy to improve cancer patients’ quality of life [1, 2]. An overall prevalence of herbal formulas use is estimated from 13 to 63% among cancer patients in the United States [3]. Herbal medicine can enhance the response rate or chemosensitivity, reduce side-effects of chemotherapy, and prolong the survival time of cancer patients [4–6]. To date, a number of herbs and herbal formulas as an adjuvant have been reported to be beneficial to cancer patients, such as PHY906 (Huangqin Tang) [7], Liu Jun Zi Tang (Rikkunshito in Japanese) [8], and Shaoyao Gancao decoction (shakuyaku-kanzo-to in Japanese) [9].

Shaoyao Gancao decoction (SGD), a classical analgesic prescription, is composed of Shaoyao (*Paeoniae Radix Alba*) and Gancao (*Glycyrrhizae Radix et Rhizoma*) in the ratio of 1:1. It is originated from *the Treatise on Febrile Diseases*, and is widely used in Asia to relieve menstrual pain, muscle spasm, and muscle pain [10, 11]. In Japan and China, doctors prefer to prescribe SGD with chemotherapeutic agent paclitaxel to relieve paclitaxel-induced myalgia and arthralgia [9, 12], and painful peripheral neuropathy [13, 14]. However, in view of the narrow therapeutic window of chemotherapeutic agents, this concomitant use increases the risk of clinically relevant herb–antineoplastic agent interactions [15]. While reducing the toxic side effects of paclitaxel, SGD also increases the metabolism of paclitaxel [15]. Our previous study has proved that pretreatment with SGD for 14 consecutive days significantly decreases the area under the curve and increases the total clearance of intravenous paclitaxel in rats [15]. Most known herb-drug interactions are due to changes in metabolic routes, which is related to altered expression or functionality of drug metabolic enzymes and/or drug transporters [3, 16, 17]. Therefore, it is quite necessary to evaluate effects of SGD on drug metabolic enzymes and drug transporters.

Cytochrome P450 (CYP450) occupies an important role in the metabolism and detoxification process of drug and other endo-or xeno-biotics. The largest fraction of the CYP450 CYP3A4, representing 40% of the total hepatic and 80% of the total intestinal CYPs [18, 19], is involved in the metabolism of approximately 50% of marketed drugs [20]. Drug transporter multidrug resistance protein 1 (MDR1) encodes P-glycoprotein, an efflux pump, and then plays an important role in the absorption and presystemic elimination of many xenobiotics [21]. Furthermore, the regulation of CYP3A4 and MDR1 is mediated primarily through the activation of nuclear xenobiotic pregnane X receptor (PXR) [22, 23]. The ethanol extracts of Shaoyao or Gancao have been shown to activate human PXR and induce CYP3A4 reporter constructs in HepG2 cells [24]. The aqueous extracts of Shaoyao or Gancao have been reported to induce P- glycoprotein-mediated drug transport [23, 25, 26]. Therefore, we speculate that the mechanism underlying the interaction from SGD might be related to regulation of CYP3A4 and/or MDR1 expression via PXR pathway.

**Fig. 1:**
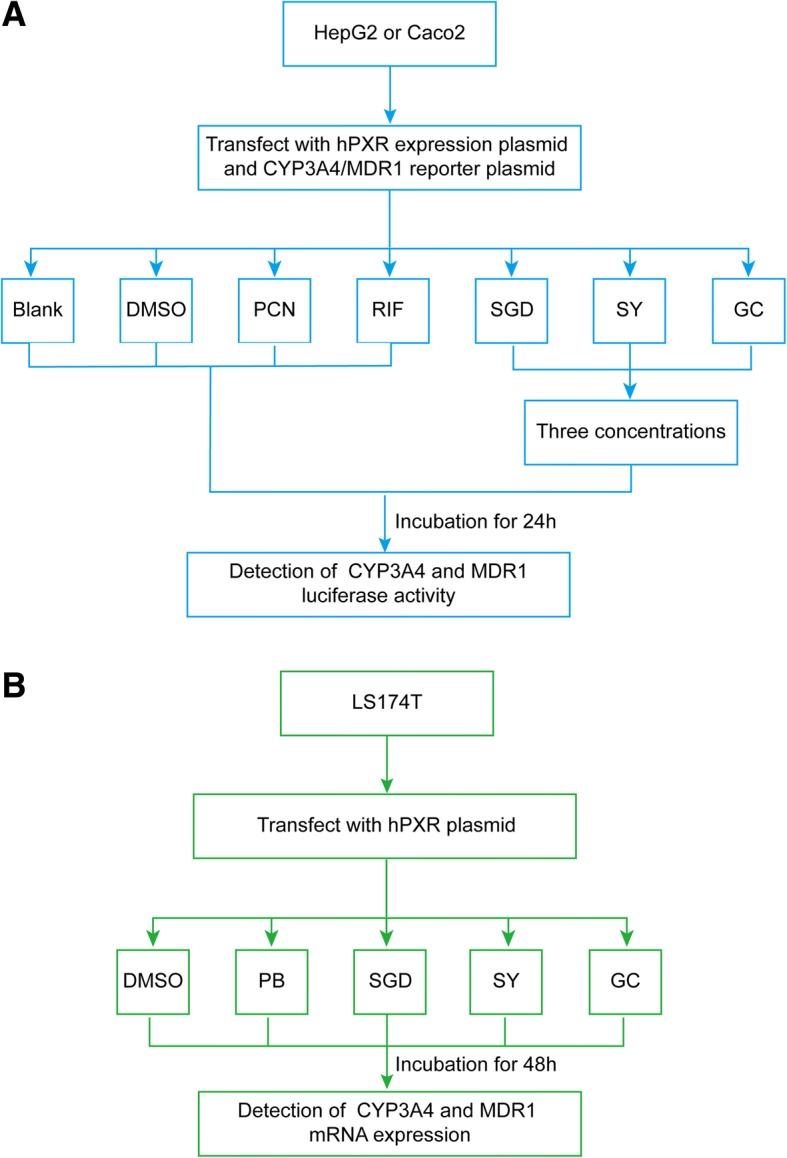
The workflow of the present study. **a** The workflow of the study that whether SGD regulates CYP3A4 or MDR1 luciferase activities through hPXR. HepG2 and Caco2 cells were co-transfected with a PXR-expressed CYP3A4 or MDR1 dual luciferase reporter gene constructs and then exposed to a range of SGD, or Shaoyao (SY), or Gancao (GC). After 24 h, luciferase activities were evaluated. **b** The workflow of the study that whether SGD regulates CYP3A4 or MDR1 mRNA expression through hPXR. LS174T cells were co-transfected with PXR plasmid and then exposed to SGD, or Shaoyao (SY), or Gancao (GC). After 48 h, CYP3A4 and MDR1 mRNA expression were measured

In this study, human hepatoma cell line HepG2 and colon carcinoma cell lines Caco2 were used to determine the potential of SGD to induce PXR-mediated transcriptional regulation of CYP3A4 and MDR1 *in vitro*. Cells were co-transfected with a PXR-expressed CYP3A4 or MDR1 dual luciferase reporter gene constructs and then exposed to a range of SGD, or Shaoyao (SY), or Gancao (GC). Luciferase activities were determined using a Dual-luciferase reporter assay kit. In addition, the effect of SGD on CYP3A4 and MDR1 mRNA expression in PXR-transfected LS174T cells was measured using real-time qPCR. The present study also investigated the contribution of constituent herbs Shaoyao and Gancao to SGD. The workflow is illustrated in Fig. 1.

## Methods

### Chemicals

Rifampicin (RIF), pregnenolone 16α-carbonitrile(PCN), phenobarbital (PB), dimethyl sulfoxide (DMSO), diethyl pyrocarbonate (DEPC) were purchased from Sigma-Aldrich (St. Louis, MO, USA). RIF, PCN, and PB were dissolved in DMSO at concentrations appropriate for the specific studies in which they were used. Trypsin and Taq DNA polymerase were purchased from Gibco BRL (Grand Island, NY, USA). Reverse transcription system, dual-luciferase reporter assay system, pGL4.17-Luc and pGEM-T constructs were purchased from Promega (Madison, WI, USA).

### Preparation of SGD, SY and GC

Shaoyao (*Paeoniae Radix Alba*, the root of *Paeonia lactiflora* Pall.) and Gancao (*Glycyrrhizae Radix et Rhizoma*, the root and rhizome of Glycyrrhiza uralensis Fisch.) were purchased from Xiangya Hospital of Central south university (Changsha, China). The crude herbs were authenticated by Prof. SY Hu, Department of Chinese Herbal Medicine of Central South University. Voucher specimens (No. 20140505 and No. 20140506) were deposited at the Laboratory of Ethnopharmacology in Xiangya Hospital. Crude drugs of SGD (SY: GC, 1:1, *w*/*w*) 200 g were immersed in 400 ml distilled water for 30 min and boiled for 40 min, then filtered. The residue was boiled and filtered once more. The filtrates from each time were combined and centrifuged at 3000 rpm for 30 min at room temperature. The supernatant was filtered through a 0.22-μm filter. The concentration of the original SGD was adjusted to 500 mg crude drug per milliliter, and then diluted into 0.1, 1, 2 mg/ml. SY (*Paeoniae Radix Alba* 100 g) or GC (*Glycyrrhizae Radix et Rhizoma* 100 g) was immersed in 400 ml distilled water, and processed as SGD. The concentration of the original SY and GC was adjusted to 250 mg crude drug per milliliter, and then diluted into 0.05, 0.5, 1 mg/ml. Decoctions were maintained at 4 °C for further use.

Meanwhile, six key compounds (gallic acid, albiflorin, paeoniflorin, glycyrrhizin, benzoic acid and liquiritigenin) from SGD for quality control using a Waters Acquity ultra-performance liquid chromatography (UPLC) system have been reported in our previous studies [27–29]. Among the six compounds, gallic acid, albiflorin, paeoniflorin and benzoic acid are for SY, while glycyrrhizin and liquiritigenin are for GC.

### Cell culture

Three cell lines were used in this study including HepG2 (human Caucasian hepatocellular carcinoma), Caco2 (human colon adenocarcinoma) and LS174T (human colon adenocarcinoma). Three cell lines were purchased from the Shanghai Institute of Cell Biology, Chinese Academy of Sciences (Shanghai, China). The HepG2 line was maintained in Dulbecco’s modified Eagle medium (DMEM) high glucose medium (HyClone), Caco2 and LS174T lines were maintained in DMEM/F12 medium (HyClone), at 37 °C in a humidified atmosphere containing 5% CO_2_. All media were supplemented with 10% fetal bovine serum (FBS, Gibco), added with 100 U/ml penicillin and 100 U/ml streptomycin (Beyotime, Shanghai, China). The medium was replaced every 3 days.

### Plasmids construction

The expression plasmid for the human PXR receptor pcDNA3.1-PXR was kindly provided by Prof. Guo Wang (Institute of Clinical Pharmacology, Central South University, Changsha, China), containing the full-length human PXR [30].

The CYP3A4-Luc reporter constructs containing the basal promoter (− 362 ~ + 53 bp) with proximal PXR response element and the distal xenobiotic responsive enhancer module (− 7836 ~ − 7208 bp) of the CYP3A4 gene 5′-flanking region inserted to pGL4.17-Basic reporter vector has been reported previously [30]. The construction of MDR1-Luc reporter gene vector has been described previously [21, 30]. Human MDR1 promoter fragment (− 7975 ~ − 7013 bp) containing the cluster of xenobiotic-responsive enhancer modules was amplified by PCR with primers 5′- TCTGCTAGCAGTGTTTCTTGT-3′, containing a natural *NheI* site, and 5′-AATCTCGAGCATATAAGGCAACTGTTTTGT-3′, introducing an *Xho*I site. The *NheI*/*Xho*I-digested PCR fragment was ligated between the *NheI*/*Xho*I sites of pGL4.17-Basic luciferase reporter vector.

### Cell transient transfections

Transient transfections of HepG2 and Caco2 cells were performed as described previously [30]. In brief, prior to cell transfection for 24 h, HepG2 or Caco2 cells were seeded in 24-well and 6-well plates at a density of 1 × 10^5^ cells per well. Transfections were performed in triplicate using 600 ng CYP3A4/MDR1-Luc, 100 ng pcDNA3.1-PXR (or 100 ng pcDNA3.1), 10 ng internal control PRL-SV40, and 5 μl lipofectamine 2000 (Invitrogen) in a well. Four to six hours after transfection, HepG2 cells were washed in DMEM, replaced with DMEM + 10% FBS medium and treated with drugs 24 h later, while Caco2 cells were washed in DMEM/F12, replaced with DMEM/F12 + 10% FBS medium and treated with drugs 24 h later.

LS174T cells (1.2 × 10^5^ per well) were seeded into 24-well and 6-well plates and cultivated for 24 h. Then LS174T cells were transfected with 100 ng/well PXR expression plasmids or pcDNA3.1 vector or weren’t transfected. Four to six hours after transfection, LS174T cells were washed in DMEM/F12, replaced with DMEM/F12 + 10% FBS medium and treated with drugs 24 h later.

### Western blot analysis

After transfection, we firstly detected expression of PXR protein in non-transfected, vector-transfected, and PXR-transfected cells by western blot. As for HepG2 or Caco2 cells in 6-well plates, we set non-transfected cells as the blank group, cells transfected with 600 ng pGL4.17-CYP3A4 (or MDR1) + 100 ng pcDNA3.1 + 10 ng PRL-SV40 as the vector group, cells transfected with 600 ng pGL4.17-CYP3A4 (or MDR1) + 100 ng pcDNA3.1-PXR + 10 ng PRL-SV40 as the trans-PXR group. As for LS174T cells in 6-well plates, we set non-transfected cells as the blank group, cells transfected with 100 ng pcDNA3.1 + 10 ng PRL-SV40 as the vector group, cells transfected with 100 ng pcDNA3.1-PXR + 10 ng PRL-SV40 as the trans-PXR group.

Western blot analysis was performed according to the standard protocol. Following 48 h transfection, transfected cells were washed with cold phosphate buffer saline once and lysed using 50 μl radioimmunoprecipitation assay lysis buffer (Pierce; Thermo Fisher Scientific, Inc.) according to the manufacturer’s protocol. After 30 min on ice, lysates were clarified by centrifugation at 12000 × rpm for 15 min at 4 °C. Amounts of protein 50–100 μg were loaded on a 10% SDS-PAGE and subsequently transferred to polyvinylidene difluoride membranes. Following a blocking incubation with TBST containing 5% non-fat dried milk at room temperature for 2 h, membranes were incubated at 4 °C overnight with primary antibody (1:1000 dilution) to PXR (rabbit, no. 15607–1-AP, Proteintech) or β-actin (mouse, no. 60008–1-Ig, Proteintech), followed by incubation at room temperature for 1 h with the goat anti-mouse or anti-rabbit horseradish peroxidase conjugated secondary antibody (1,3000 dilution; cat no. ab97051, Proteintech). The membranes were analyzed using ECL detection reagent (Thermo). β-actin was used as an internal control. All experiments were conducted in triplicate.

### Herbal treatment in HepG2 or Caco2 cells and detecting luciferase activity

Co-transfected HepG2 or Caco2 cells were treated with rifampicin (10 μM), PCN (10 μM), SGD (0.1, 1, and 2 mg/ml), SY (0.05, 0.5, and 1 mg/ml), GC (0.05, 0.5, and 1 mg/ml), or the solvent control (0.1% DMSO) for 24 h, respectively. Vector control (VC) group was transfected with pcDNA3.1-PXR + PRL-SV40 + pGL4.17 (or pcDNA3.1 + PRL-SV40 + pGL4.17-CYP3A4) and treated with 0.1% DMSO. The blank group was co-transfected with pcDNA3.1-PXR + PRL-SV40 + pGL4.17-CYP3A4 and treated with nothing. The firefly luciferase activity was detected using the dual-luciferase reporter assay (Promega) according to the manufacturer’s protocol. The Renilla luciferase activity was used as an internal control. Each experiment was repeated three times in triplicate. The efficiency of transfection in each treatment groups was adjusted by the ratio of firefly luciferase activity to Renilla luciferase activity.

### Real-time qPCR analysis of CYP3A4 and MDR1 mRNA in LS174T

To determine CYP3A4 and MDR1 mRNA induction, LS174T cells were transfected with 100 ng/well PXR expression plasmids or pcDNA3.1 vector or weren’t transfected. Following 24 h transfection, appropriate cell samples were exposed to phenobarbital (1 mM), SGD (1 mg/ml), SY (1 mg/ml), GC (1 mg/ml) or the solvent control (0.1% DMSO) for 48 h, respectively. Then cells were harvested and the mRNA levels of CYP3A4, MDR1 were measured by real-time qPCR.

Total RNA isolation was performed using TRIzol reagent (Invitrogen) and converted into cDNA using the Fermentas RT kit according to the manufacturer’s instructions. The primers designed using Primer Premier 5.0 are presented in Table 1. PCR was performed in a total reaction volume of 30 μL, containing 15 μL 2 × SYBR Green PCR Master Mix (Applied Biosystems), 3 μL cDNA, 1 μL forward primer (10 μM), 1 μL reverse primer (10 μM), and 10 μL double-distilled water. Real-Time qPCR analysis was performed using the Applied Biosystems 7500 real-time PCR system. The temperature profile was 95 °C for 10 min; 40 cycles of 95 °C for 10 s, 59 °C for 50 s; melting curve program 60–95 °C. Real-time qPCR for each gene of interest was performed in triplicate, and the expression values were calculated as their average. Gene expression was evaluated in at least three independent experiments and each performed in triplicates. The expression of the target genes were normalized to the reference gene β-actin and then processed using the 2^−ΔΔCt^ method [31]. The data are expressed as the fold changes in the activation of gene expression relative to the non-transfected group with 0.1% DMSO treatment (set to be 1).

**Table 1:** Primer sequences used for real-time qPCR

Gene	Orientation	5′-3′ sequence
*CYP3A4*	Forward	5’-GCACCGAGTGGATTTCCTT-3′
	Reverse	5’-GGACATCAGGGTGAGTGGC-3’
*MDR1*	Forward	5’-AGGCTCGCCAATGATGC-3’
	Reverse	5’-TCCTGTCCCAAGATTTGCTAT-3’
*β-actin*	Forward	5’-CATCCTGCGTCTGGACCTGG-3’
	Reverse	5’-TAATGTCACGCACGATTTCC-3’

### Statistical analysis

All data are expressed as means ± S.E.M of three independent experiments performed in triplicates. The data were first tested for the normality of distribution with Shapiro-Wilk test. The distribution of samples is approximately normal. Differences between groups were analyzed using a one-way ANOVA with Welch correction, followed by the Games Howell test. All statistical analyses were performed using SPSS 23.0 (International Business Machines Corp., Armonk, NY, USA). Values of *p* < 0.05 are considered to be statistically significant.

## Results

### SGD and its constituent herbs significantly enhance the CYP3A4 promoter activities via hPXR in HepG2 and Caco2 cells

**Fig. 2:**
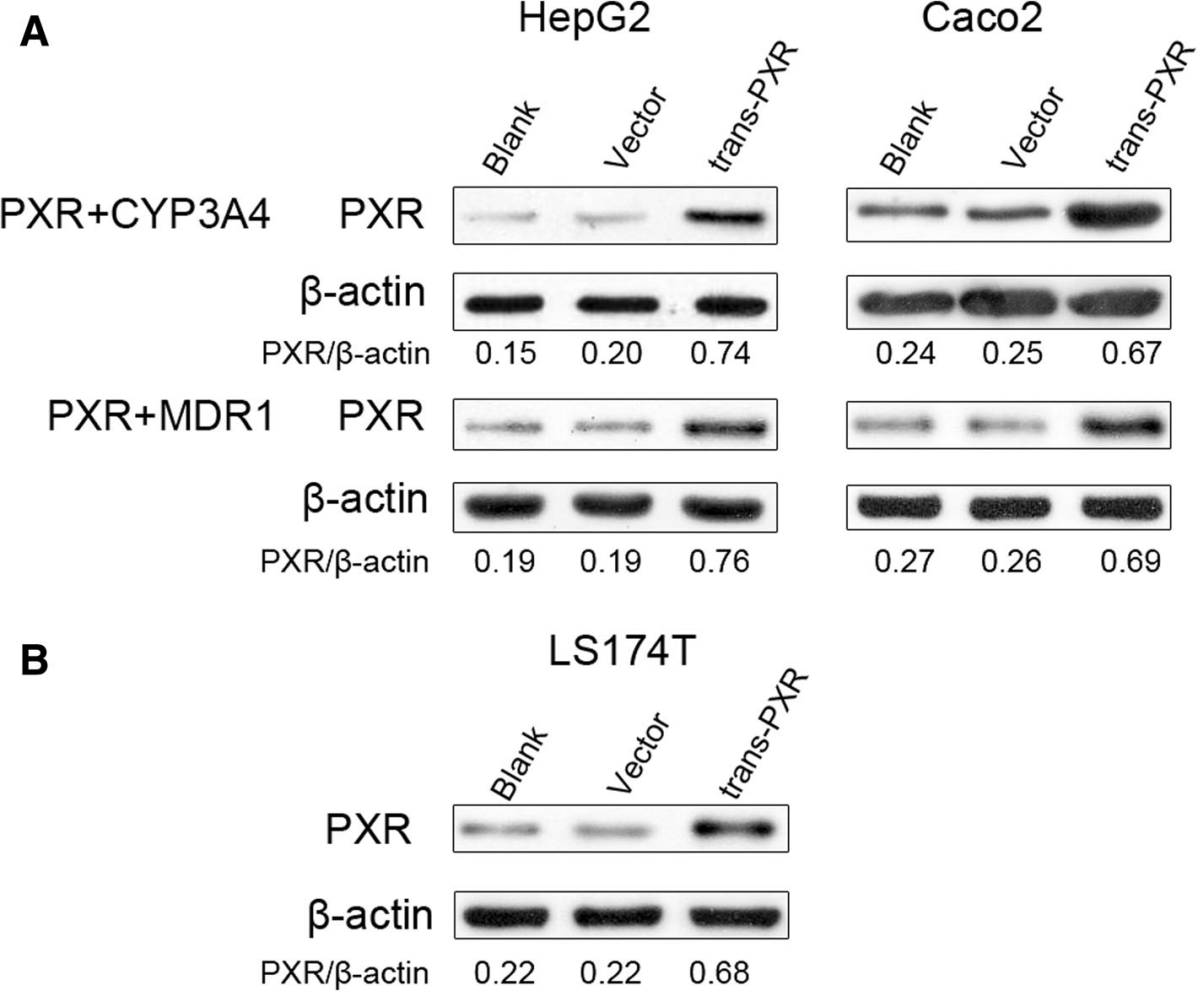
The expression of PXR fusion protein *in vitro* was detected by Western blotting. **a** Blank: cells were non-transfected, Vector: cells were transfected with 600 ng CYP3A4 (or MDR1) + 100 ng pcDNA3.1 + 10 ng PRL-SV40, trans-PXR: cells transfected with 600 ng CYP3A4 (or MDR1) + 100 ng pcDNA3.1-PXR + 10 ng PRL-SV40. **b** Blank: cells were non-transfected, Vector: cells were transfected with 100 ng pcDNA3.1 + 10 ng PRL-SV40, trans-PXR: cells were transfected with 100 ng pcDNA3.1-PXR + 10 ng PRL-SV40

First, we examined whether SGD and its constituent prescriptions (SY, GC) affect the activation of CYP3A4-Luc reporter constructs through hPXR. Since CYP3A4 is mainly distributed in the liver and intestine organs, we used the two human tumor cell lines [32]. We successfully transfected PXR-CYP3A4 plasmid constructs to HepG2 and Caco2 cells (Fig. 2). Twenty-four hours following co-transfection, HepG2 and Caco2 cells were separately treated with rifampicin (10 μM, hPXR agonist), PCN (10 μM, rodent PXR agonist), 0.1, 1, 2 mg/ml SGD or 0.05, 0.5, 1 mg/ml constituent prescriptions (SY and GC) for 24 h, then detected dual luciferase activity.

**Fig. 3:**
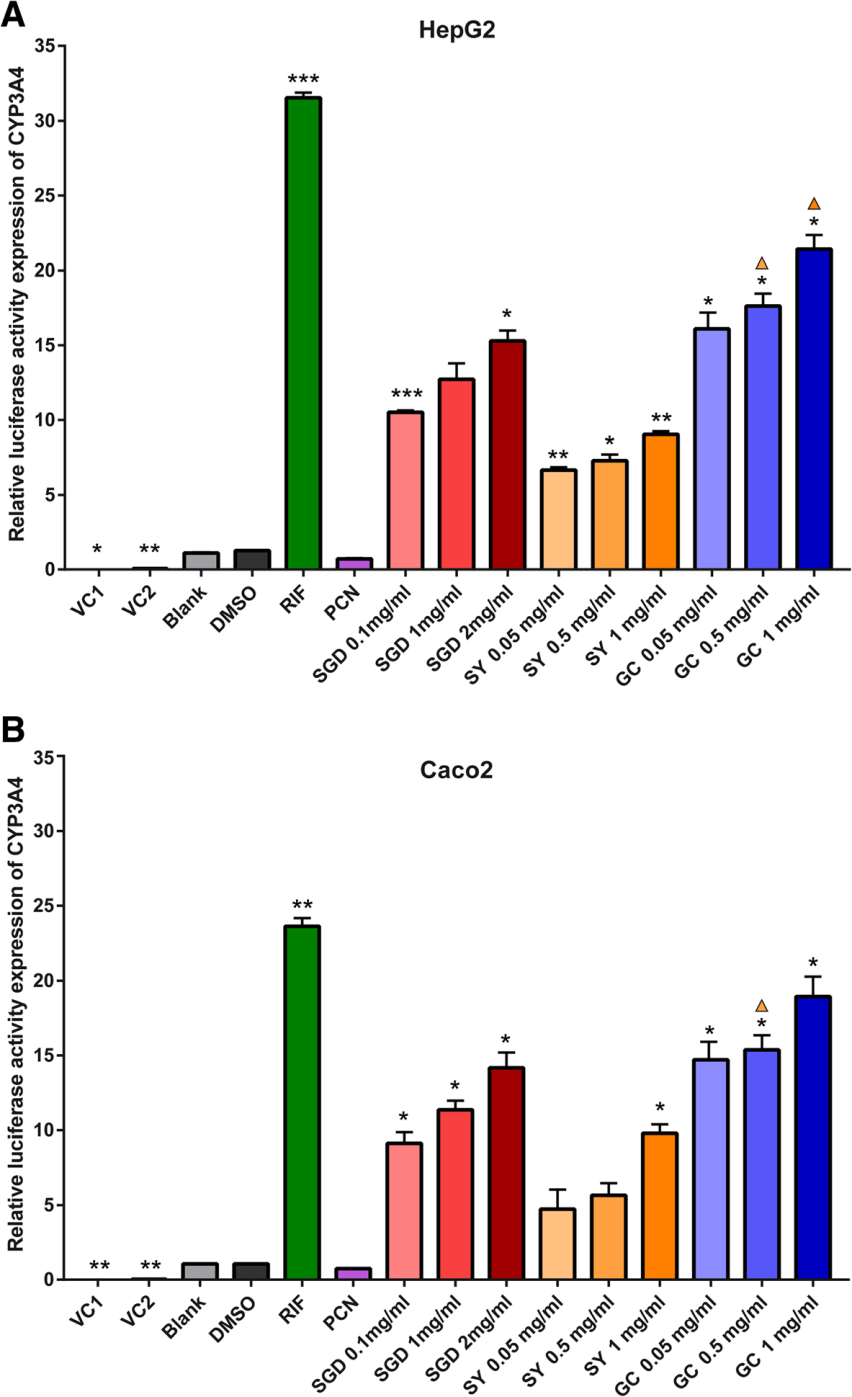
Induced effects of CYP3A4 promoter mediated via PXR by SGD, SY and GC in HepG2 (**a**) and Caco2 (**b**). There is a significant increase in positive control RIF group and most prescriptions groups when compared with 0.1% DMSO treatment group (*p* < 0.05). There is no significant decrease in negative control PCN group when compared to the DMSO group (*p* > 0.05). VC1: cells were transfected with pcDNA3.1-PXR, PRL-SV40, pGL4.17; VC2: cells were transfected with pcDNA3.1, PRL-SV40, pGL4.17-CYP3A4; other groups: cells were transiently transfected with pcDNA3.1-PXR, PRL-SV40, pGL4.17-CYP3A4. All data were obtained from three independent experiments performed by triplicate and expressed as mean ± S.E.M. Statistical significance was determined by one-way ANOVA with Welch correction, followed by the Games Howell test for pairwise comparisons. One-way ANOVA with Welch’s correction revealed significant differences among the groups (Welch’s *p* < 0.001). *p < 0.05, **p < 0.01, ***p < 0.001 compared with the DMSO group; ▲*p* < 0.05 represents GC group compared to SY group with the same concentration

Results of specific activity values of various concentrations groups are presented in Fig. 3. In HepG2 and Caco2 cells co-transfected with hPXR expression plasmid, rifampicin, SGD and its constituent prescriptions significantly increase the luciferase activities of CYP3A4 when compared with 0.1% DMSO vehicle control group (*p* < 0.05). In HepG2 cells transfected with PXR expression plasmid, rifampicin (10 μM), SGD (0.1 and 2 mg/ml), SY (0.05, 0.5, and 1 mg/ml) and GC (0.05, 0.5, and 1 mg/ml) markedly increase CYP3A4 luciferase activity by 24.80, (8.27, 12.05), (5.24, 5.74, 7.12), (12.68, 13.86, 16.85)-fold compared with DMSO, respectively. While 1 mg/ml SGD increase 10.00-fold CYP3A4 luciferase activity to the DMSO group without a statistical significance. In Caco2 cells transfected with PXR expression plasmid, CYP3A4 luciferase activity is significantly increased by rifampicin (22.26), SGD (8.62, 10.75, 13.40), 1 mg/ml SY (9.24), GC (13.87, 14.53, 17.83) when compared with DMSO, respectively. The increases in CYP3A4 luciferase activities by three decoctions show a concentration-dependent trend.

However, in both transfected HepG2 and Caco2 cells, PCN treatment doesn’t cause a significant increase in CYP3A4 luciferase activities when compared with the DMSO group. Previous studies have found rifampicin activates human but not rodent PXR, while PCN activates rodent but not human PXR [33, 34]. Consistent with the previous report [35], similar activation of hPXR and inductions of CYP3A4 and MDR1 by rifampicin are observed in HepG2, Caco-2 cells, except PCN.

### SGD and its constituent herb GC markedly elevate the MDR1 promoter activities via hPXR in HepG2 and Caco2 cells

Then, we examined whether the three decoctions induce the activation of MDR1-Luc reporter constructs through hPXR in HepG2 and Caco2 cells. We successfully transfected PXR-MDR1 plasmid constructs in HepG2 and Caco2 cells (Fig. 2). Twenty-four hours following transfection, HepG2 and Caco2 cells were separately treated with rifampicin (10 μM, hPXR agonist), PCN (10 μM, rPXR agonist), SGD (0.1, 1, and 2 mg/ml), SY (0.05, 0.5, and 1 mg/ml) and GC (0.05, 0.5, and 1 mg/ml) for 24 h, detected dual luciferase activity.

**Fig. 4:**
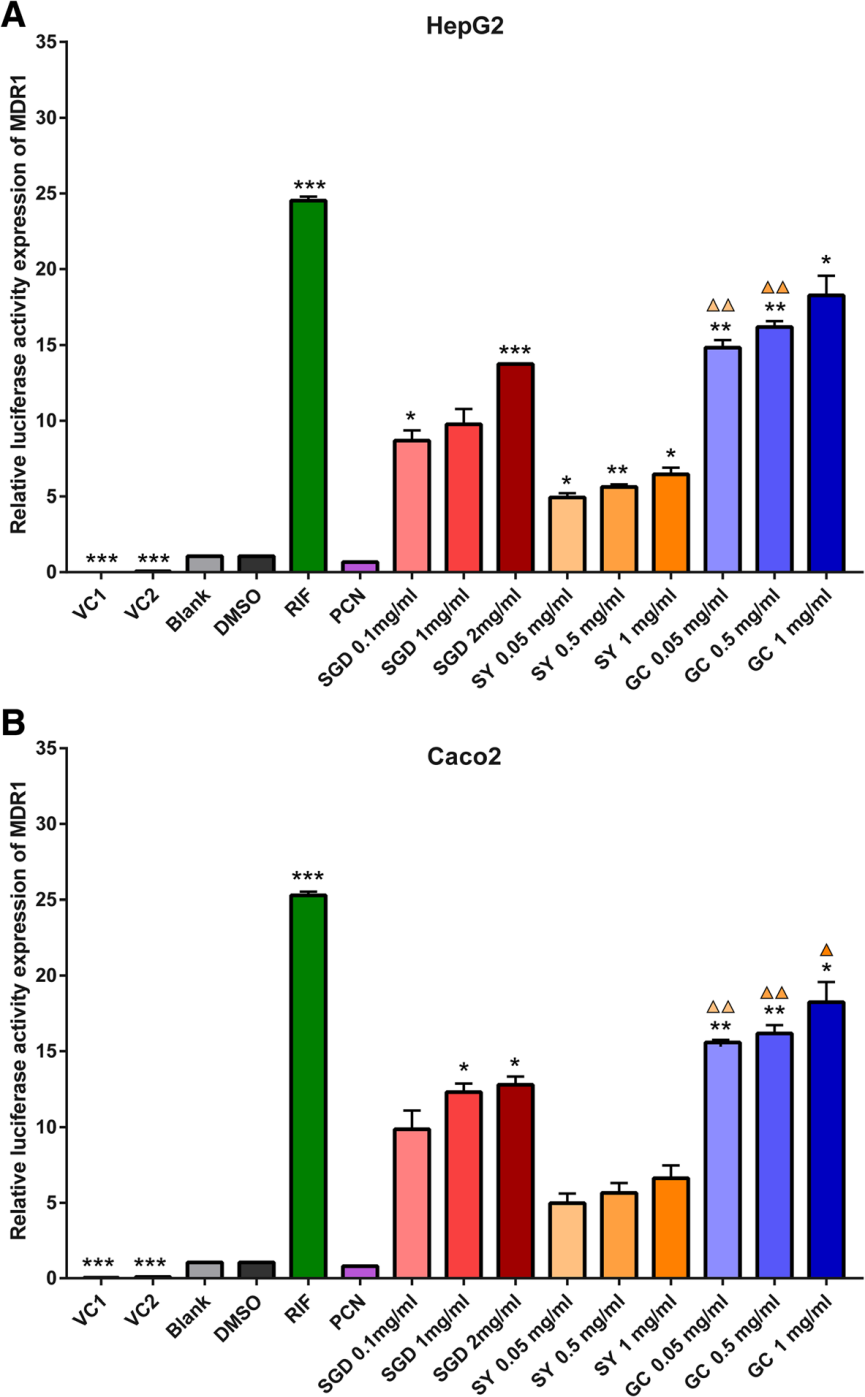
Induced effects of MDR1 promoter mediated via PXR by SGD, SY and GC in HepG2 (**a**) and Caco2 (**b**). There is a significant increase in positive control RIF group and most prescriptions groups when compared with 0.1% DMSO treatment group (*p* < 0.05). There is no significant decrease in negative control PCN group when compared to the DMSO group (*p* > 0.05). VC1: cells were transfected with pcDNA3.1-PXR, PRL-SV40, pGL4.17; VC2: cells were transfected with pcDNA3.1, PRL-SV40, pGL4.17-CYP3A4; other groups: cells were transiently transfected with pcDNA3.1-PXR, PRL-SV40, pGL4.17-CYP3A4. All data were obtained from three independent experiments performed by triplicate and expressed as mean ± S.E.M. Statistical significance was determined by one-way ANOVA with Welch correction, followed by the Games Howell test. One-way ANOVA with Welch’s correction revealed significant differences among the groups (Welch’s *p* < 0.001). *p < 0.05, **p < 0.01, ***p < 0.001 compared with the DMSO group; ▲*p* < 0.05, ▲▲*p* < 0.01 represents GC group compared to SY group with the same concentration

Results are shown in Fig. 4. In hPXR-transfected HepG2 and Caco2 cells, rifampicin, SGD and its constituent prescription GC significantly increase the luciferase activities of MDR1 when compared with 0.1% DMSO group (*p* < 0.05). However, SY only shows the significant increase in luciferase activities of transfected HepG2 cells. The increases of the three decoctions in MDR1 luciferase activities also show a concentration-dependent trend. In PXR-HepG2 cells, MDR1 luciferase activity is significantly increased by rifampicin (22.90), 0.1 mg/ml SGD (8.10), 2 mg/ml SGD (12.80), SY (4.60, 5.25, 6.04), GC (13.83, 15.14, 17.10) when compared with DMSO, respectively. In PXR-Caco2 cells, the luciferase activity of MDR1 is significantly increased by rifampicin (24.10), 1 mg/ml SGD (11.71), 2 mg/ml SGD (12.19) and GC (14.86, 15.43, 17.33) when compared with DMSO. However, in HepG2-hPXR and Caco2-hPXR cells, PCN treatment doesn’t cause a significant increase in MDR1 luciferase activities when compared with the DMSO group.

In a word, in HepG2 and Caco2 cells, SGD and its constituent prescription GC significantly increase promoter activities of CYP3A4 and MDR1 via PXR in a concentration-dependent manner. While SY doesn’t significantly increase promoter activities of MDR1 in transfected Caco2 cells. Like rifampicin, 1 mg/ml GC is a strong agonist of PXR, followed by SGD, and SY ranges the weakest.

### SGD and its constituent herbs upregulate expression of CYP3A4 and MDR1 mRNA in LS174T via PXR

**Fig. 5:**
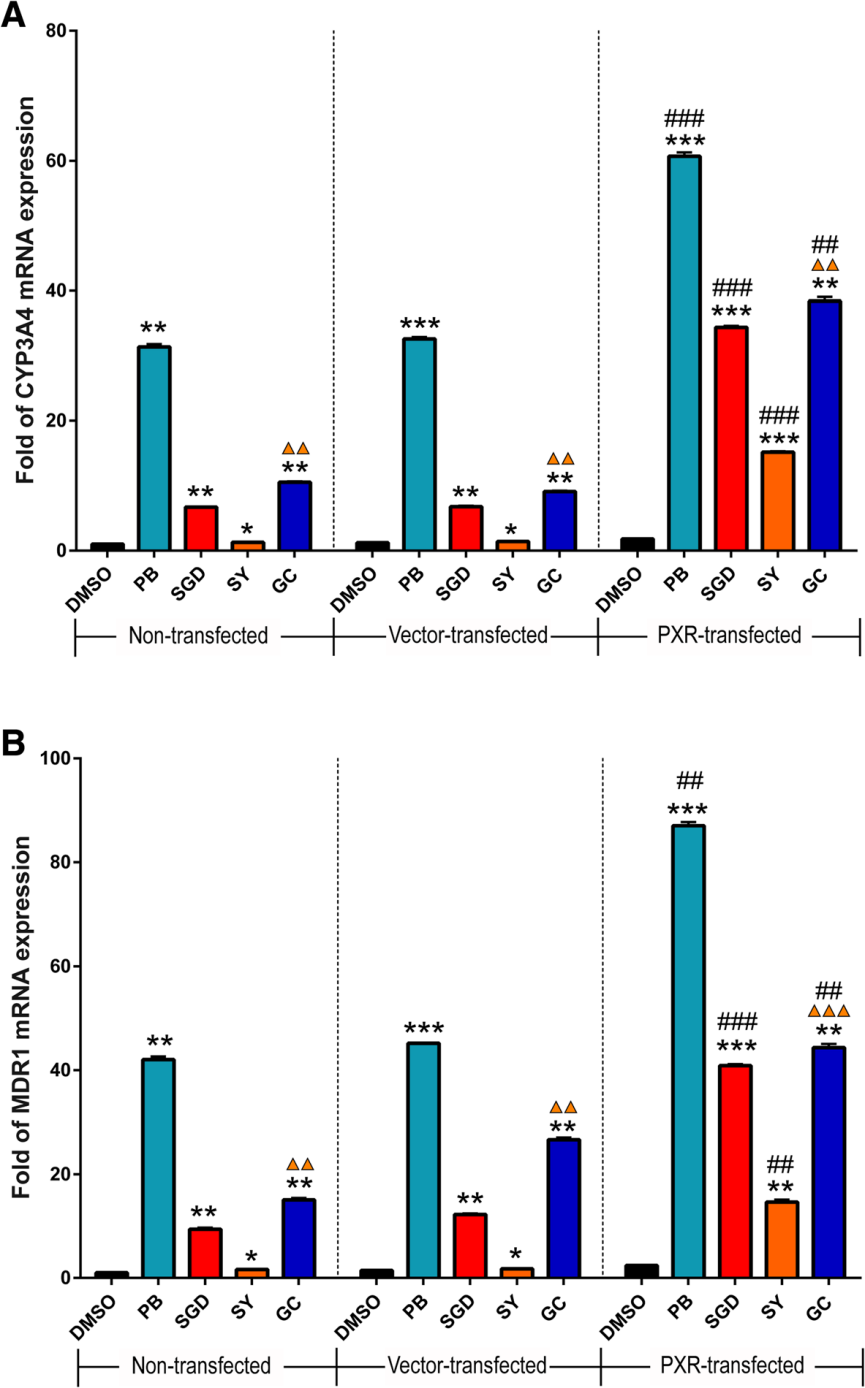
The up-regulation of phenobarbital (PB), SGD, SY, GC on CYP3A4 (**a**) and MDR1 (**b**) mRNA expression. LS174T cells were transfected with 400 ng/well pcDNA3.1-PXR, or pcDNA3.1 vector, or weren’t transfected. Cells were then exposed to phenobarbital (1 mM), or SGD (1 mg/ml), or SY (1 mg/ml), or GC (1 mg/ml) for 48 h. mRNA expression of CYP3A4 and MDR1 was determined using real-time qPCR. Data are presented as -fold increase to non-transfected group with DMSO treatment. Data are presented as means ± S.E.M of three independent experiments performed in triplicates. Statistical significance was determined by one-way ANOVA with Welch correction, followed by the Games Howell test. One-way ANOVA with Welch’s correction revealed significant differences among the groups (Welch’s *p* < 0.001). *p < 0.05, **p < 0.01, ***p < 0.001 compared to the DMSO group with the same transfection; ▲▲*p* < 0.01, ▲▲▲*p* < 0.001 represents GC group compared to SY group with the same transfection; ##*p* < 0.01, ###*p* < 0.001 compared to vector-transfected group with the same drug treatment

Finally, to verify effects of SGD and its constituent herbs on CYP3A4 and MDR1 mRNA expression levels by PXR activation, we used PXR-transfected LS174T cell lines for real-time qPCR (Fig. 5). LS174T cells express endogenous hPXR with inducible activity [32], which was supported by our observation of a low PXR protein expression in LS174T (Fig. 2B). Twenty-four hours following transfection, LS174T cells were exposed to phenobarbital (1 mM, PXR agonist), 1 mg/ml SGD, SY and GC for 48 h, separately. In order to avoid the effect of herbal concentration, the same concentration was adopted. In LS174T cells with non-transfected or blank vector-transfected, phenobarbital, SGD, SY and GC significantly up-regulate level of CYP3A4 and MDR1 mRNA compared with corresponding DMSO groups (*p* < 0.05). And GC has a significant up-regulation when compared to SY (*p* < 0.01). Significantly enhancements of CYP3A4 and MDR1 mRNA expression are also observed in PXR-transfected cells. This indicates phenobarbital, SGD, SY and GC may activate endogenous and exogenous PXR and then significantly up-regulate CYP3A4 and MDR1 mRNA expression in LS174T cells. In PXR-transfected groups, increases of CYP3A4 and MDR1 mRNA expression are significantly higher by phenobarbital, SGD, SY, GC than them in non-transfected groups or vector-transfected groups (*p* < 0.01). All of that means SGD and its constituent prescriptions up-regulate expression of CYP3A4 and MDR1 mRNA in LS174T via PXR.

## Discussion

**Fig. 6:**
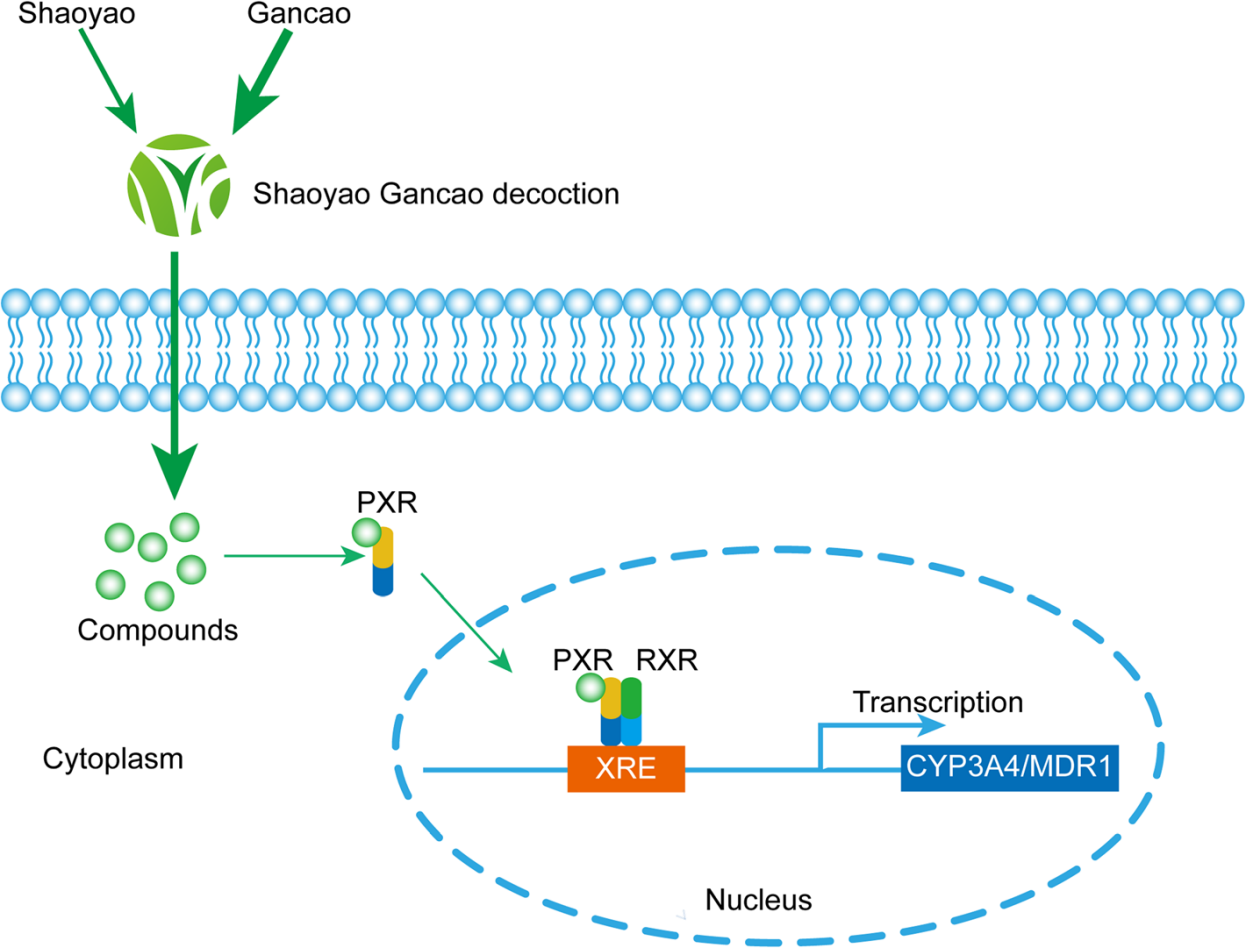
The illustration of the present work. PXR translocates from the cytoplasm to the nucleus of the cells when bound to and activated by ligands, then activated PXR binds to target genes’ DNA xenobiotic response elements (XREs) as a heterodimer or heterotetramer with the retinoid X receptor (RXR). Then induce the transcription of the target genes. PXR could be activated by SGD, and then bind to XREs of CYP3A4 and MDR1, induce the transcription of them, finally affect the metabolism of other drugs

The present study demonstrates that SGD, SY and GC result in a concentration-dependent increase in CYP3A4 and MDR1 promoter activity in HepG2 and Caco2 cells via PXR activation after 24 h exposure. Moreover, we show that SGD, SY and GC are capable of up-regulating CYP3A4 and MDR1 gene expression in LS174T through activation of PXR pathway. Interestingly, Gancao makes the main contribution to SGD on the induction. The illustration is shown in Fig. 6.

Chinese herbal medicine has been shown to have synergistic actions with chemotherapy and reduce their side effects [5]. An overall prevalence of combined utilization is estimated up to 63% among cancer patients in the United States [3]. However, with the increasing co-administration of herbal medicine with chemotherapy, herb-drug interactions have become an important issue in clinical practice. It has been found that traditional herbal formulas cause clinically-relevant interactions and affect the metabolism and pharmacokinetics of chemotherapeutic agents [3, 36, 37]. In the previous study, we observed that pretreatment with SGD for 14 days significantly altered pharmacokinetics of paclitaxel involved in decreasing the area under the curve and increasing the total clearance of intravenous paclitaxel in rats [15]. In general, the reduction in area under the curve and increase of the clearance after co-administration is due to the induction of the metabolic enzyme of drugs and/or the drug transporters [20]. However, studies of SGD on drug metabolic enzymes and drug transporters are rare so far. Therefore, it is urgent to evaluate effects of SGD on drug metabolic enzymes and drug transporters and to uncover the underlying molecular mechanism.

The CYP450 enzyme system is responsible for the biotransformation of more than 90% of the drugs on the market [38]. The inducers of CYP450 can speed up the metabolism of substrates and drugs, including most chemotherapeutic drugs [30]. And the largest fraction of the P450 reactions is catalyzed by CYP3A4 [18], which is mainly distributed in the liver and intestine and metabolizes at least 50% of marketed pharmaceutical agents [39]. MDR1 encodes P-glycoprotein, an important transmembrane efflux pump protein, to limit the absorption of drugs and contribute to the excretion of drugs in the intestine [32]. The induction of many CYPs and drug transporters occurs by a similar mechanism, where ligand activation of PXR [34]. PXR could be activated by ligands and translocate from the cytoplasm to the nucleus [34]. Then activated PXR binds to specific promoter responsive elements of many xenobiotic enzymes and transporter genes as a heterodimer or heterotetramer with the retinoid X receptor (RXR), thus, induces the transcription [40–42]. Here, we established SGD and its constituent herbs (SY and GC) as natural potent agonists of PXR and proved that they induce the transcription of intestinal and hepatic CYP3A4 and MDR1 via PXR activation *in vitro*. Those *in vitro* results might partially account for the pharmacokinetics alteration of drugs via SGD *in vivo*.

SGD, an antispasmodic herbal formula, is consisted of Shaoyao (*Paeoniae Radix Alba*) and Gancao (*Glycyrrhizae Radix et Rhizoma*). It is widely used in Asia to treat muscle cramps, many kinds of pain, abdominal spasmodic pain, and dysmenorrhea [10]. The previous study has shown that the combination of Shaoyao and Gancao might work “synergistically” to improve the efficacy or reduce the toxicity of individual components [43]. The contribution of constituent herbs should also be assessed. In the present study, the induction of CYP3A4 and MDR1 by SY and GC is in accordance with previous reports [25, 26, 44, 45]. The present study also showed the induction of CYP3A4 and MDR1 via Gancao was much higher than Shaoyao. That indicates Gancao is the main contributor to SGD on the induction of CYP3A4 and MDR1. Excess of SGD or Gancao can also lead to a failure of most drugs since they enhance the drug metabolizing enzymes which could lead to a rapid removal of the drugs before its therapeutic action. SGD or Gancao should be administrated with caution.

In most cases, it is not good for PXR to be activated. Conversely, the activation of PXR by herbs may have favorable effects. Recent studies have revealed much more complex regulatory pathways governed by PXR, such as lipid and glucose metabolism, bile acid metabolism and inflammation [34]. PXR activation prevents lithocholic acid-induced hepatotoxicity, suggesting that PXR agonists may be useful in the treatment of human cholestatic liver disease [46]. A strong inducer of PXR St. John’s wort significantly affects serum concentrations of irinotecan, a chemotherapeutic agent, consequently reducing the antineoplastic effect and toxicity of this CYP3A4 substrate [47]. Overall, these studies highlight the dual features of PXR activation: promoting drug metabolism leads to serious potential drug interactions and treatment failures, and activation of the detoxification system helps protect our body from invading of toxic substances. Therefore, when prescribing herbs with agents, it is necessary to monitor the plasma concentration of agents to guarantees effectiveness and reduce toxicity.

The limitations of the present study are clear, and further explorations in the future are demanded. Firstly, our study only focuses on the activation of PXR. Beyond PXR, more nuclear receptors, such as CAR, are needed to be studied in the future. Besides, we illustrate the up-regulation of SGD on CYP3A4 and MDR1 via PXR activation, we didn’t evaluate effects of active compounds from SGD.

## Conclusions

This study firstly observed SGD induce intestinal and hepatic CYP3A4 and MDR1 promoter and enhance mRNA expression via activating PXR pathway *in vitro*. And Gancao plays a predominant role in the induction effects. This study highlights a potential of SGD on the PXR-mediated regulation of drug metabolic enzymes and drug transporters.

## Abbreviations

CYP450: Cytochrome P450
DEPC: Diethyl pyrocarbonate
DMEM: Dulbecco’s modified eagle medium
DMSO: Dimethyl sulfoxide
FBS: fetal bovine serum
GC: Gancao
MDR1: Multidrug resistance protein 1
PB: Phenobarbital
PCN: Pregnenolone 16α-carbonitrile
PXR: Pregnane X receptor
RIF: Rifampicin
SGD: Shaoyao Gancao decoction
SY: Shaoyao
UPLC: Ultra-performance liquid chromatography
VC: Vector control
